# 12-Lipoxygenase governs the innate immune pathogenesis of islet inflammation and autoimmune diabetes

**DOI:** 10.1172/jci.insight.147812

**Published:** 2021-07-22

**Authors:** Abhishek Kulkarni, Annie R. Pineros, Melissa A. Walsh, Isabel Casimiro, Sara Ibrahim, Marimar Hernandez-Perez, Kara S. Orr, Lindsey Glenn, Jerry L. Nadler, Margaret A. Morris, Sarah A. Tersey, Raghavendra G. Mirmira, Ryan M. Anderson

**Affiliations:** 1 Center for Diabetes and Metabolic Diseases and Department of Pediatrics, Indiana University School of Medicine, Indianapolis, Indiana, USA.; 2 Kolver Diabetes Center and Department of Medicine, The University of Chicago, Chicago, Illinois, USA.; 3 Department of Medicine, Eastern Virginia Medical School, Norfolk, Virginia, USA.; 4 Department of Medicine, New York Medical College, Valhalla, New York, USA.

**Keywords:** Autoimmunity, Endocrinology, Diabetes, Islet cells, Macrophages

## Abstract

Macrophages and related myeloid cells are innate immune cells that participate in the early islet inflammation of type 1 diabetes (T1D). The enzyme 12-lipoxygenase (12-LOX) catalyzes the formation of proinflammatory eicosanoids, but its role and mechanisms in myeloid cells in the pathogenesis of islet inflammation have not been elucidated. Leveraging a model of islet inflammation in zebrafish, we show here that macrophages contribute significantly to the loss of β cells and the subsequent development of hyperglycemia. The depletion or inhibition of 12-LOX in this model resulted in reduced macrophage infiltration into islets and the preservation of β cell mass. In NOD mice, the deletion of the gene encoding 12-LOX in the myeloid lineage resulted in reduced insulitis with reductions in proinflammatory macrophages, a suppressed T cell response, preserved β cell mass, and almost complete protection from the development of T1D. 12-LOX depletion caused a defect in myeloid cell migration, a function required for immune surveillance and tissue injury responses. This effect on migration resulted from the loss of the chemokine receptor CXCR3. Transgenic expression of the gene encoding CXCR3 rescued the migratory defect in zebrafish 12-LOX morphants. Taken together, our results reveal a formative role for innate immune cells in the early pathogenesis of T1D and identify 12-LOX as an enzyme required to promote their prodiabetogenic phenotype in the context of autoimmunity.

## Graphical abstract

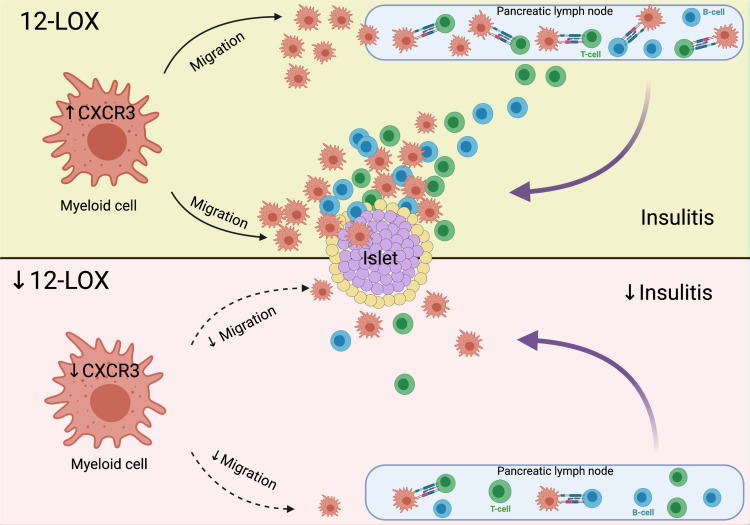

## Introduction

The 2 major forms of diabetes, type 1 (T1D) and type 2 (T2D), represent disorders of glucose homeostasis whose common feature is the failure of the islet β cell to secrete adequate insulin (1). The underlying etiologies of β cell failure in each form of diabetes differ. In the case of T1D, an active dialog between β cells and cells of the immune system results in the cytokine-induced dysfunction of β cells in the early phase of disease, and in the destruction of β cells by autoreactive T cells in the later phase (2). A pathology that characterizes this dialog is “insulitis,” a feature in which islets are invaded by cells of the myeloid and lymphoid lineage (3). Insulitis and the nature and timing of cells invading the islet have been characterized best in the NOD mouse model of T1D (3, 4). Insulitis has also been demonstrated in human T1D, albeit at a much lower frequency than that in NOD mice (5). Drug targeting of the immune system has been the foundational approach in clinical studies attempting to prevent or reverse T1D, but these studies have targeted the activation or function of lymphoid cell responses with variable and transient effects (6).

An emerging perspective in T1D posits that islet inflammation arising from innate immune responses may initiate the formation of neoepitopes in β cells, thereby signaling a cascade of immune signaling events leading to the development of T cell–mediated autoimmunity (7). Central to this perspective are cells of the myeloid lineage, which include macrophages and DCs. These cells mediate inflammation and present antigen to the adaptive immune system (8) and have been observed in the early stages of insulitis in mouse models of T1D (9–11). The presence of “resident” macrophages and their closely related antigen-presenting DCs have been demonstrated in early insulitic lesions in NOD mice (9, 12), and their functional inhibition via antibody-mediated or clodronate-mediated global sequestration slows or prevents the occurrence of T1D (11, 13–16). However, as a strategy for T1D, the therapeutic depletion of macrophages would be undesirable given the consequences on overall immunity. Nevertheless, the identification of amenable targets that function primarily in myeloid cells during the pathogenesis of T1D would complement existing lymphoid cell–targeting strategies.

Lipoxygenases (LOX) are enzymes that catalyze the dioxygenation of polyunsaturated fatty acids. Specifically, 12/15-LOX (referred to henceforth as simply 12-LOX), which is predominantly expressed in macrophages and pancreatic islets in mice (17), catalyzes the conversion of arachidonic acid to the eicosanoids 12-hydroxyeicosatetraenoic (12-HETE) and 15-hydroxyeicosatetraenoic acid (15-HETE) (18). Global deletion of the gene encoding 12-LOX in mice (*Alox15*) on the NOD background results in near-total protection of both sexes from T1D development, with a striking reduction in insulitis and the early accumulation of macrophages (19); similarly, delivery of a small molecule inhibitor of 12-LOX (ML351) in NOD mice shortly after the development of insulitis protects against progression of insulitis and glycemic deterioration (20). Whereas these findings provide evidence for the safety and efficacy of targeting 12-LOX in the context of T1D, they leave unclarified the specific cell of action and molecular mechanisms of 12-LOX. In this study, we leveraged genetic models in zebrafish and mice to investigate the role of 12-LOX in the myeloid cell pathogenesis of T1D. Our findings provide evidence for a determinative role for myeloid 12-LOX in the initiation of T1D and highlight the seminal role of innate immunity in the propagation of T1D autoimmunity.

## Results

### Development of a zebrafish platform to interrogate the role of macrophages in the early pathogenesis of T1D.

**Figure 1:**
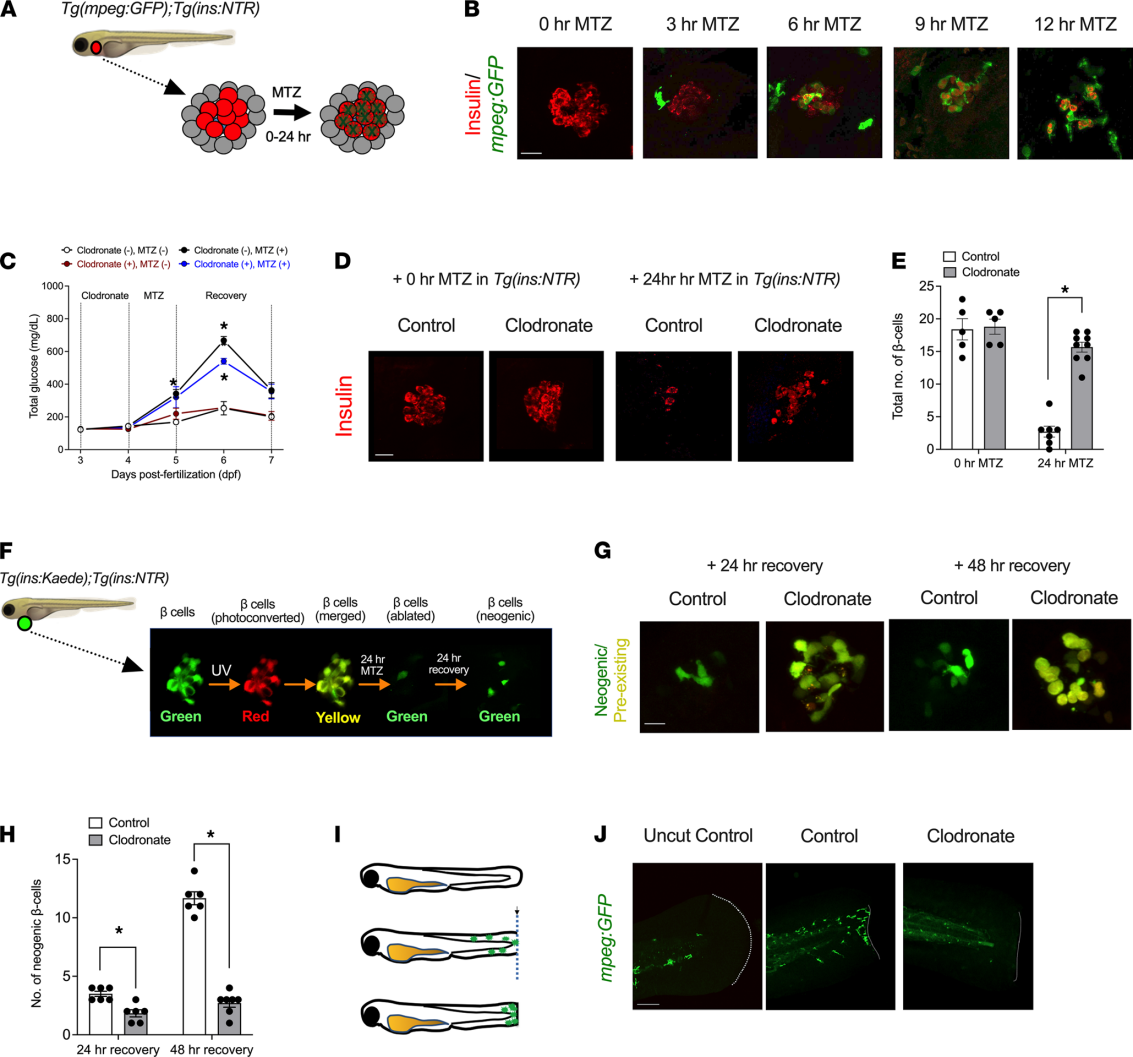
Macrophages promote β cell loss and hyperglycemia after injury in zebrafish. (**A**) Schematic representation of the β cell injury assay in transgenic *Tg(mpeg:eGFP*);*Tg*(*ins:NTR*) zebrafish at 3 dpf, wherein islet β cells (red) are selectively destroyed upon incubation with MTZ, with the concomitant/subsequent entry of macrophages (green). (**B**) Representative images of islets from zebrafish treated for the times indicated with MTZ, then stained for insulin (β cells, red) and GFP (macrophages, green). Scale bar: 10 μm. (**C**) Free glucose measurements of whole zebrafish lysates, treated as indicated in the panel. *n* = 3–5 lysates per condition (20 fish per lysate), and *P < 0.05 (by 1-way ANOVA with post hoc Tukey’s test) for the corresponding values compared with untreated controls (no clodronate, no MTZ). (**D**) Representative images of islets from zebrafish stained for insulin (β cells, red) under the conditions indicated. Scale bar: 10 μm. (**E**) Quantitation of β cell number from the experiment represented in **D** (*P < 0.05 by unpaired 2-tailed *t* test). (**F**) Schematic representation of the β cell regeneration assay, where photoconversion of *Kaede* protein results in red+green (= yellow) preexisting β cells and newly formed (neogenic) β cells enter as green cells. (**G**) Representative images of islets from zebrafish exhibiting preexisting (yellow) and neogenic (green) β cells at 24 hours and 48 hours of recovery after MTZ treatment under the conditions (control or clodronate) indicated. Scale bar: 10 μm. (**H**) Quantitation of neogenic β cell number from the experiment represented in **G** (*n* = 6–7 fish/condition; *P < 0.05 by unpaired 2-tailed *t* test). (**I**) Schematic representation of the zebrafish tailfin injury assay, where tailfins of *Tg*(*mpeg:GFP*) fish at 3 dpf are mechanically cut with a blade, and the migration of macrophages (green) are observed at the site of injury. (**J**) Representative tailfin images of uninjured and injured zebrafish tails stained with GFP (macrophages, green) under the conditions indicated. Dotted line shows the tailfin injury site. Scale bar: 100 μm. In all panels, data are presented as mean ± SEM. GFP, green fluorescent protein; NTR, nitroreductase; *Tg(mpeg:eGFP*), transgenic fish containing enhanced GFP–labeled macrophages; *Tg*(*ins:NTR*), transgenic β cell NTR–expressing zebrafish; MTZ, metronidazole.

The zebrafish is a powerful model organism with which to test hypotheses and mechanisms that can then be interrogated further in mammalian systems. Developing zebrafish (3–4 days post fertilization [dpf]) exhibit a discrete pancreatic islet with functional β cells (21) and intact innate immunity with *mpeg*-expressing macrophages (22). We utilized a model of chemically induced β cell oxidative injury that we described previously (23), in which zebrafish harboring β cell–specific expression of the gene encoding bacterial nitroreductase (NTR) are treated with the prodrug metronidazole (MTZ). We crossed transgenic β cell NTR–expressing zebrafish [*Tg*(*ins:NTR*)] with transgenic fish containing enhanced green fluorescent protein–labeled (eGFP-labeled) macrophages [*Tg*(*mpeg:eGFP*)] to generate a double-transgenic fish line that allows for chemical β cell injury and simultaneous visualization of macrophages in real-time. Upon treatment of double-transgenic 3 dpf zebrafish with 7.5 mM MTZ (Figure 1A), we observed a rapid loss (within 12 hours) of β cells with the influx of macrophages and evidence of β cell phagocytosis (Figure 1B). Glucose levels increased within 24 hours after MTZ treatment compared with untreated control fish, whose levels remained unchanged (Figure 1C). To demonstrate that β cell damage and loss were at least partially attributable to macrophage activity, we repeated the MTZ ablation experiment after transpericardial injection with 5 mg/mL clodronate liposomes at 3 dpf. Clodronate sequesters and depletes macrophages in animal models, including zebrafish (24, 25). Within 24 hours of clodronate liposome injection, we observed a near-complete depletion of macrophages, whereas the control empty liposomes did not affect the abundance of the macrophages (Supplemental Figure 1A; supplemental material available online with this article; https://doi.org/10.1172/jci.insight.147812DS1). In clodronate-injected MTZ-treated fish, we observed a significant reduction in hyperglycemia compared with only MTZ-treated fish (Figure 1C) and preservation of β cell number compared with control-injected fish (Figure 1, D and E). These data suggest that our MTZ model in zebrafish mimicked the early phases of T1D, where inflammation driven by macrophages contributes to the development of hyperglycemia (13, 14).

Because zebrafish β cells have a capacity for rapid regeneration, we asked if the apparent preservation of β cell number upon the depletion of macrophages was either a reflection of surviving preexisting β cells after MTZ treatment or their rapid replacement by neogenesis. To distinguish between these possibilities, we utilized the *Tg*(*ins:Kaede^s949^*) transgenic zebrafish line, which drives the expression of a green photoconvertible protein and thus permits the pulse labeling of β cells with green and/or UV-converted red fluorescence (Figure 1F). In this transgenic line, preexisting β cells will be labeled yellow after photoconversion (combined red and green), whereas neogenic β cells formed after the UV exposure will be labeled green (Figure 1F). We generated double-transgenic [*Tg*(*ins:NTR*);(*ins:Kaede*)] zebrafish and then treated them with MTZ to induce β cell stress. When β cells were injured, the control-injected fish showed β cell neogenesis at 24 hours of recovery that increased 3-fold by 48 hours of recovery (Figure 1, G and H). By contrast, at both 24 hours and 48 hours after β cell ablation, clodronate-injected fish showed significantly decreased β cell neogenesis relative to controls, with no increase at 48 hours (Figure 1, G and H). These data indicate that β cell numbers were greater in the fish lacking macrophages because of the persistence of preexisting β cells rather than β cell neogenesis.

To ensure that our macrophage tissue injury model was not peculiar to MTZ treatment, we also performed mechanical tailfin injury assays in 3 dpf *Tg(mpeg:eGFP)* zebrafish. In the tailfin injury assay (shown schematically in Figure 1I), macrophages rapidly migrate to the injury site as part of an inflammatory response (26). After the injury, macrophages were observed to migrate to the site of tissue injury, as expected, whereas in clodronate-injected fish, macrophages were not observed at the site of injury (Figure 1J). Taken together, the data in Figure 1 support an inflammatory model in zebrafish, where β cell injury is affected, in part, by macrophages and where macrophage depletion reduces β cell loss and mitigates hyperglycemia.

### 12-LOX was required for macrophage-directed β cell injury.

**Figure 2:**
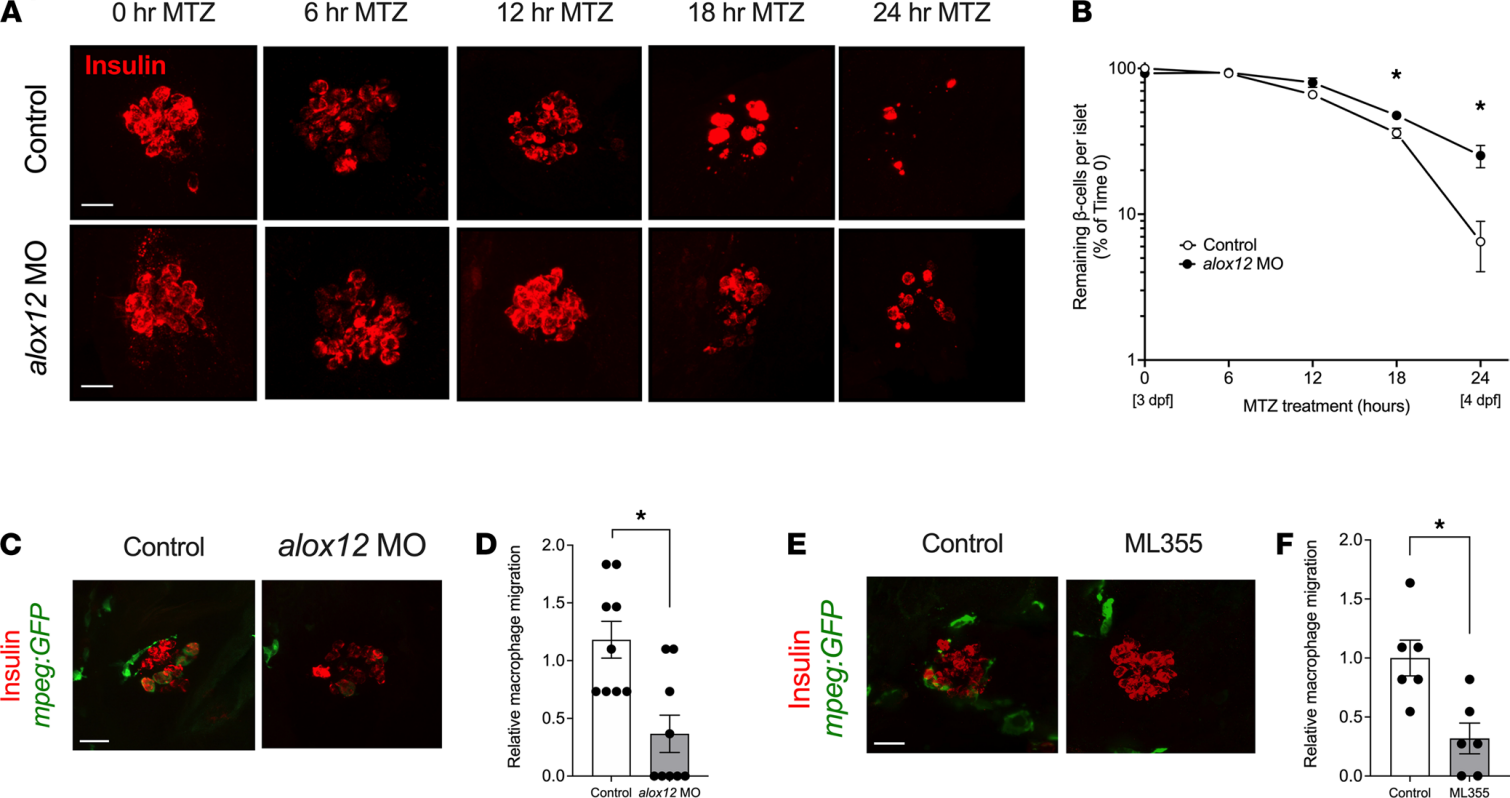
Depletion or inhibition of 12-LOX protects against β cell loss in zebrafish. Zebrafish were treated with *alox12* MO or the 12-LOX inhibitor ML355 prior to treatment with MTZ at 3 dpf. (**A**) Representative images of islets from zebrafish stained for insulin (β cells, red). (**B**) Quantification of β cell number in pancreatic islets of fish (expressed as % of β cells remaining relative to time 0) after MTZ treatment. *n* = 6–8 fish per time point. *P < 0.05 (by unpaired 2-tailed *t* test) for the time points indicated compared with control-treated fish. (**C**) Representative images of islets from control and *alox12* MO zebrafish stained for insulin (β cells, red) and GFP (macrophages, green) at 24 hours after MTZ treatment. (**D**) Quantification of the relative number of macrophages located at the site of injured islets from the experiment shown in **C** (*n* = 9 fish per condition; *P < 0.05 by unpaired 2-tailed *t* test). (**E**) Representative images of islets from control-treated and ML355-treated zebrafish stained for insulin (β cells, red) and GFP (macrophages, green). (**F**) Quantification of the relative number of macrophages located at the site of injured islets from the experiment shown in **E** (*n* = 6 fish per condition; *P < 0.05 by unpaired 2-tailed *t* test). Scale bar: 10 μm. In all panels, data are presented as mean ± SEM. 12-LOX, 12-lipoxygenase; MO, morpholino; MTZ, metronidazole; GFP, green fluorescent protein.

Previous studies in mouse models of T1D demonstrated that global deletion of 12-LOX reduces the early infiltration of macrophages into islets, preserves β cell mass, and prevents hyperglycemia (19). We recently demonstrated the presence of a zebrafish 12-LOX ortholog encoded by *alox12*, which exhibits similar catalytic activity and product profiles (including the production of 12-HETE) to the mouse and human enzymes (27). To test a role for 12-LOX in β cell injury mediated by macrophages, we utilized the zebrafish β cell injury model in the transgenic line *Tg*(*ins:NTR*) after the depletion of 12-LOX by injection of a translation-blocking antisense morpholino (*alox12* MO) (27). Although the use of *alox12* MO reduces β cell number slightly (by 2–3 β cells) (27), substantial β cells remain to test the effect of MTZ treatment. With MTZ treatment, there was a time-dependent reduction in β cell number, with only 6% of β cells remaining at 24 hours (Figure 2, A and B). By contrast, there was significant preservation of β cells when the fish were treated with *alox12* MO, with about 25% of β cells remaining at 24 hours after MTZ (Figure 2, A and B). Next, we utilized the double-transgenic line *Tg*(*ins:NTR*);(*mpeg:eGFP*) to track the influx of macrophages after β cell injury in the presence or absence of *alox12* MO. As expected, MTZ treatment resulted in the migration of macrophages into the islet in control-injected fish (Figure 2, C and D), whereas in *alox12* MO fish, there was a significant 2.7-fold reduction in macrophage numbers (Figure 2, C and D). The reduction of macrophages at the site of β cell injury after the *alox12* MO was likely due to a reduction of macrophage migration, rather than a reduction in the total number of macrophages, because macrophages persist elsewhere in the embryo (unlike with clodronate injection; Supplemental Figure 1B), and similar macrophage numbers were seen in the immediate vicinity of the islet in injured *alox12* MO fish compared with uninjected control fish (Supplemental Figure 1C). Notably, control MO (which does not target any specific gene) had no effect on macrophage migration compared with uninjected controls in the tailfin injury assay (Supplemental Figure 1D).

To support our findings with the *alox12* MO and to ensure that the effects observed were not off target, we next repeated these studies utilizing ML355, a small molecule inhibitor of 12-LOX (28). As shown in Figure 2, E and F, when 12-LOX was inhibited by the treatment of fish with 10 μM ML355, macrophage infiltration into the injured islets was reduced 3.1-fold relative to vehicle-treated controls. Collectively, the data in Figure 2 suggest that 12-LOX was required for macrophage-directed β cell damage in zebrafish and raise the possibility for a similar role in β cell damage in T1D.

### 12-LOX in macrophages was required for T1D progression in the mouse.

**Figure 3:**
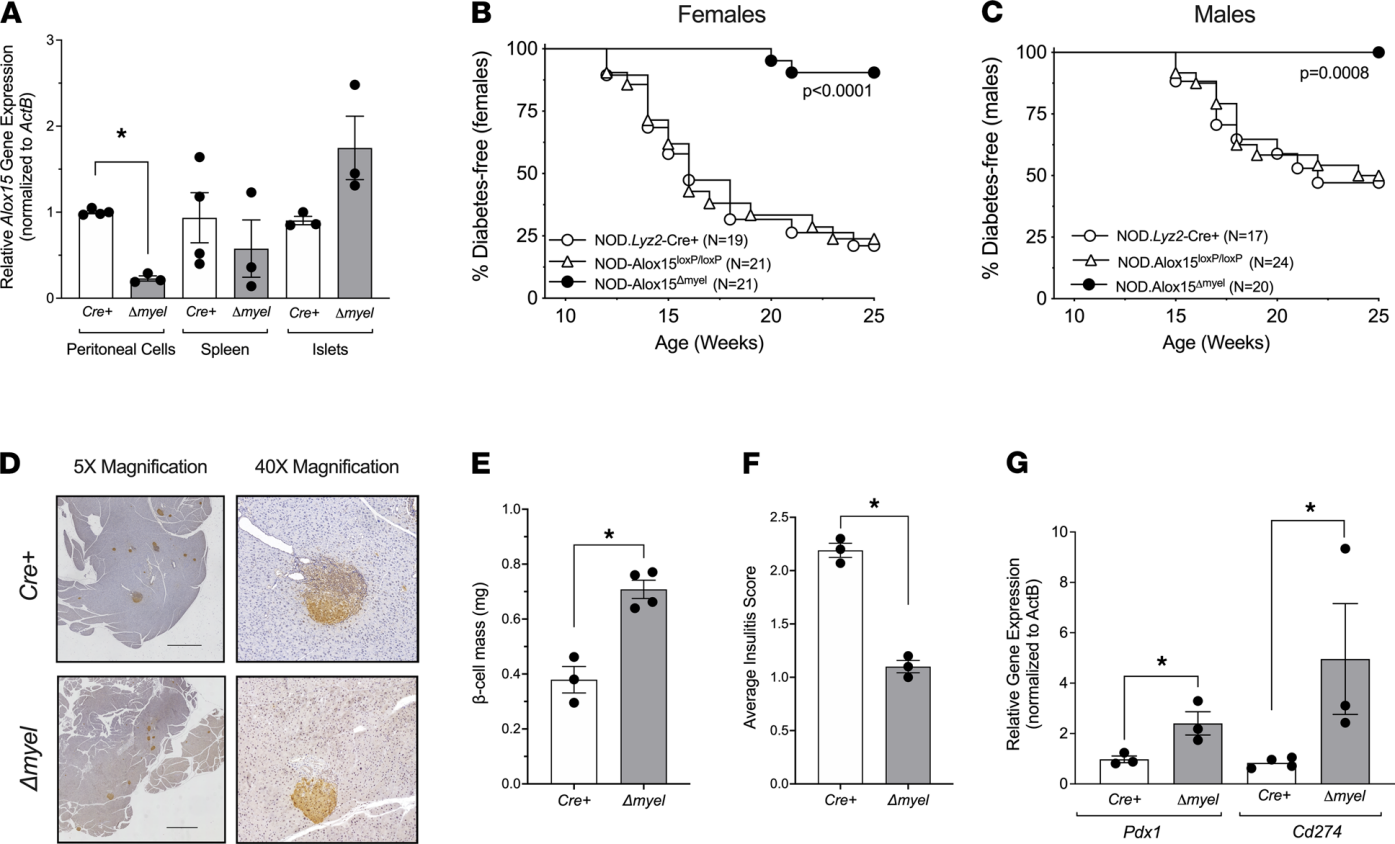
Protection from diabetes after myeloid-specific deletion of *Alox15* in NOD mice. NOD mice harboring the *Lyz2-Cre* allele were crossed to NOD mice harboring Cre recombinase sites (Loxp) flanking exons 2–5 of the *Alox15* gene to generate *NOD:Alox15*^Δmyel^** mice (Δ*myel*). *NOD:Lyz2-Cre* (Cre^+^) and *NOD:Alox15^Loxp/Loxp^* littermates were used as controls. (**A**) *Alox15* mRNA expression in peritoneal cells, spleen, and isolated islets (*n* = 3–4 per tissue); *P < 0.05 by unpaired 2-tailed *t* test. (**B**) Diabetes incidence in female mice. Number of mice per group is indicated. *P* value indicates significance by log rank test. (**C**) Diabetes incidence in male mice. Number of mice per group is indicated. *P* value indicates significance by log rank test. (**D**) Representative IHC images of mouse pancreata from *Cre*^+^ and Δ*myel* mice immunostained for insulin (brown) and counterstained with hematoxylin (blue). Scale bar: 1000 μm. (**E**) β Cell mass in *Cre*^+^ and Δ*myel* mice (*n* = 3–4 mice per genotype). (**F**) Insulitis scoring from pancreata of *Cre*^+^ and Δ*myel* mice (*n* = 3 mice per genotype). (**G**) Gene expression in isolated islets from *Cre*^+^ and Δ*myel* mice (*n* = 3 mice per genotype). For **E**–**G**, *P < 0.05 by unpaired 2-tailed *t* test. All data are presented as mean ± SEM.

To assess the applicability of our zebrafish findings to an established mammalian model of T1D, we next studied the NOD mouse model. We tested the hypothesis that 12-LOX in macrophages is responsible for the progression to T1D in NOD mice by generating a myeloid lineage–specific deletion of *Alox15* on the NOD background (*NOD:Alox15*^Δmyel^**). NOD mice harboring the *Lyz2-Cre* allele were crossed to NOD mice harboring Cre recombinase sites (Loxp) flanking exons 2–5 of the *Alox15* gene (29, 30). To test for tissue specificity of the knockout, *Alox15* mRNA was measured in peritoneal cells (containing mostly macrophages), spleen, and islets using quantitative PCR (qPCR). *NOD-Alox15*^Δmyel^** mice exhibited a significant reduction in *Alox15* expression in peritoneal cells compared with control littermates, whereas *Alox15* expression in spleen and pancreatic islets were unchanged (Figure 3A). We also confirmed the reduction of 12-LOX protein in peritoneal cells by immunoblot (Supplemental Figure 2A; see complete unedited blots in the supplemental material).

Next, we followed *NOD:Alox15*^Δmyel^** mice and control littermates (*NOD:Lyz2-Cre* and *NOD:Alox15^Loxp/Loxp^*) for the spontaneous development of T1D, defined as 2 consecutive morning blood glucose measurements of greater than 250 mg/dL. As shown in Figure 3B, whereas 75%–80% of female control littermates developed diabetes by 25 weeks of age, only 12.5% of female *NOD:Alox15*^Δmyel^** mice developed diabetes. Similar striking findings were observed in male mice: 50% of controls developed diabetes by 25 weeks of age, whereas 0% of *NOD:Alox15*^Δmyel^** developed diabetes (Figure 3C). These findings suggest that the T1D-protective phenotype previously described in global *Alox15^–/–^* mice (19) can be ascribed at least in part to its effect in macrophages.

To characterize islet pathology in NOD mice, we next performed immunostaining of pancreas sections from *NOD:Alox15*^Δmyel^** mice and *NOD:Lyz2-Cre* controls at 8 weeks of age, a time point when insulitis is established but prior to the development of T1D. As evident in the representative IHC pancreas images in Figure 3D, β cell mass was significantly increased (quantitated in Figure 3E) and the severity of insulitis was significantly reduced (quantitated in Figure 3F) in *NOD:Alox15*^Δmyel^** animals compared with *NOD:Lyz2-Cre* controls (Figure 3F). Isolated islets from *NOD.Alox15*^Δmyel^** mice revealed an increase in mRNAs levels of *Pdx1*, which encodes a key transcription factor that promotes β cell function (31), and *Cd274*, which encodes PD-L1, an immune checkpoint protein that promotes suppression of immune responses in T1D (32) (Figure 3G).

### Myeloid-specific loss of 12-LOX altered the macrophage and DC populations in pancreatic lymph nodes.

**Figure 4:**
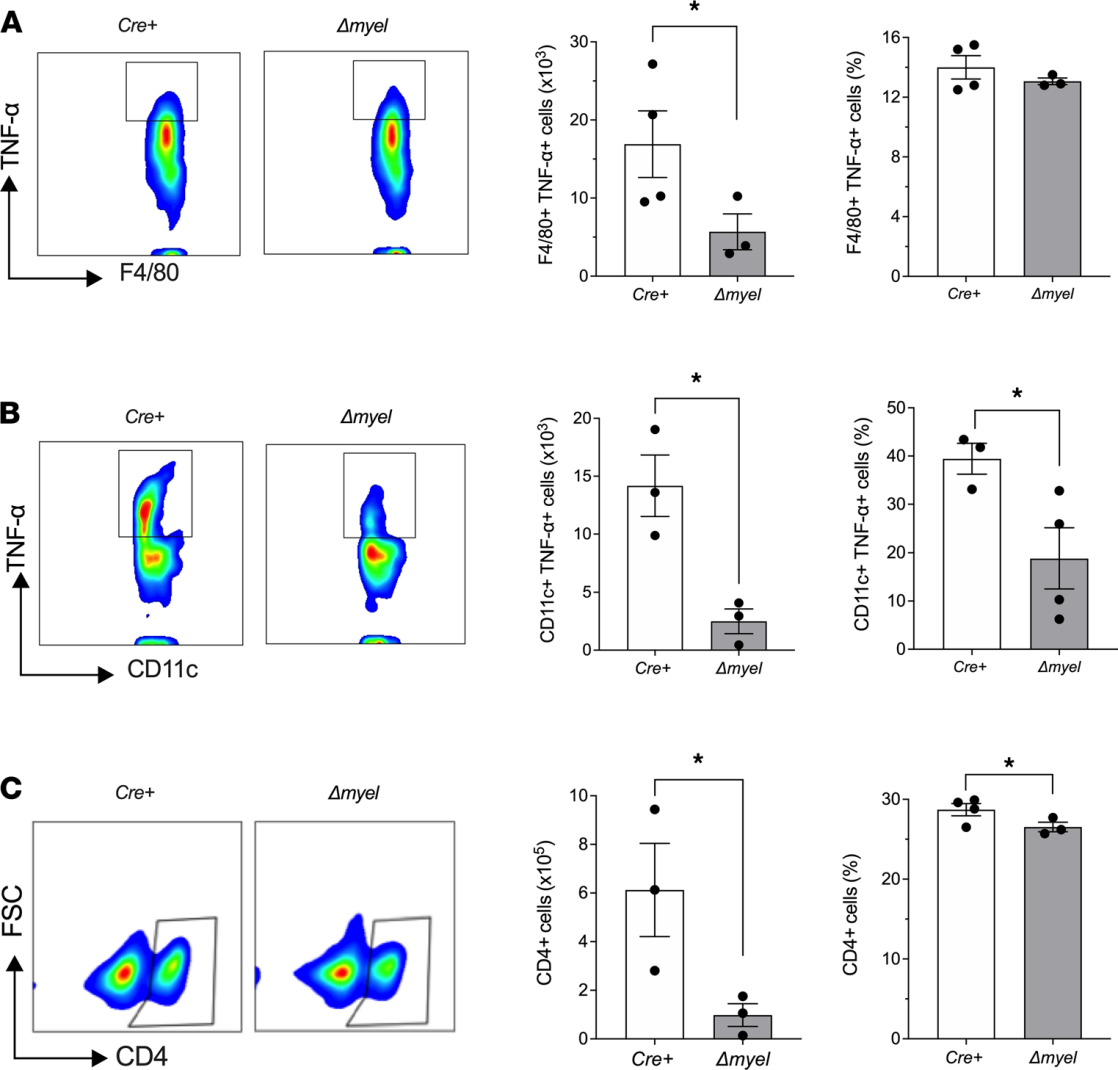
Reduced proinflammatory myeloid cell populations and CD4 T cells in pancreatic lymph nodes of *NOD:Alox15^Δmyel^* mice. Pancreatic lymph nodes were isolated from *NOD:Lyz2-Cre* (*Cre*^+^) control mice and *NOD:Alox15*^Δmyel^** (Δ*myel*) mice at 8 weeks of age and subjected to flow cytometry analysis. (**A**) Representative contour plot showing gating of F4/80^+^ TNF-α^+^ cells (left), total number of F4/80^+^ TNF-α^+^ cells (middle), and F4/80^+^ TNF-α^+^ cells as a percentage of total cells (right; *n* = 3–4 mice per genotype; *P < 0.05 by unpaired 2-tailed *t* test). (**B**) Representative contour plot showing gating of CD11c^+^ TNF-α^+^ cells (left), total number of CD11c^+^ TNF-α^+^ cells (middle), and CD11c^+^ TNF-α^+^ cells as a percentage of total cells (right; *n* = 3–4 mice per genotype; *P < 0.05 by unpaired 2-tailed *t* test). (**C**) Representative contour plot showing gating of CD4^+^ cells (left), total number of CD4^+^ cells (middle), and CD4^+^ cells as a percentage of total cells (right; *n* = 3–4 mice per genotype; *P < 0.05 by unpaired 2-tailed *t* test). All data are presented as mean ± SEM.

Pancreatic lymph nodes are a site of local antigen presentation and their immune cell composition is reflective of the nature of prevailing autoimmunity (8). To assess quantitatively if the autoimmune response was altered in *NOD:Alox15*^Δmyel^** mice, we collected pancreatic lymph nodes and performed flow cytometry for immune cell populations. Figure 4, A–C, shows that populations of proinflammatory macrophages (F4/80^+^/TNF-α^+^) and myeloid-derived antigen-presenting DCs (CD11c^+^/TNF-α^+^) were significantly reduced in the total number and as a percentage of total cells in pancreatic lymph nodes of *NOD:Alox15*^Δmyel^** compared with *NOD:Lyz2-Cre* controls. Additionally, there were selective reductions in both macrophages and DCs expressing the proinflammatory cytokine IL-1β, whereas no changes were observed in the total numbers of F4/80^+^ macrophages or CD11c^+^ DCs (Supplemental Figure 2, B–E). We also analyzed the macrophage abundance in the whole pancreas. Similar to the findings from the pancreatic lymph nodes, we observed reductions in the abundance of proinflammatory macrophages (F4/80^+^/TNF-α^+^) in pancreas, whereas the total number of macrophages remained unchanged (Supplemental Figure 2, F and G). These alterations in macrophage and DC populations were specific to the pancreatic lymph node and pancreas because these cell populations were unchanged in the spleen (Supplemental Figure 3). Because lymph nodes are a site of antigen presentation by macrophages to CD4^+^ T cells, we also quantitated CD4^+^ cells in the pancreatic lymph nodes. As shown in Figure 4C, we observed reductions in the total number of both CD4^+^ cells and CD4^+^ cells as a percentage of total cells in the pancreatic lymph nodes in *NOD:Alox15*^Δmyel^** compared with *NOD:Lyz2-Cre* controls. On further analysis of subpopulations of Th cell subsets, we observed a significant reduction in proinflammatory Th1 cells (CD4^+^IFN-γ^+^), a significant increase in Treg cells (CD4^+^Foxp3^+^), and no change in proinflammatory Th17 cells (CD4^+^IL-17^+^; Supplemental Figure 4).

### 12-LOX promoted macrophage migration.

To clarify the mechanisms underlying the less aggressive macrophages in the absence of 12-LOX, we next asked if 12-LOX governs the polarization of macrophages to a proinflammatory state. We isolated peritoneal macrophages from *Alox15^–/–^* mice or their WT littermates, performed polarization studies in vitro, then monitored phenotypes by flow cytometry. Macrophages were polarized to the classical proinflammatory “M1-like” state using a combination of LPS and IFN-γ or to the alternative antiinflammatory “M2-like” state using IL-4 (33). The macrophages from both WT and *Alox15^–/–^* mice showed indistinguishable propensity to polarize to an M1 state, as assessed by flow cytometry of M1 marker iNOS (Supplemental Figure 5A), by IL-6 secretion (Supplemental Figure 5B), and by mRNA expression of *Nos2, Il6,* and *Il12* (Supplemental Figure 5C). Similarly, upon alternative polarization, macrophages from both WT and *Alox15^–/–^* mice showed no differences by flow cytometry in the M2 marker CD206 (Supplemental Figure 6A), by IL-10 secretion (Supplemental Figure 6B) or by mRNA expression of *Arg1, Il10,* and *Tgfb* (Supplemental Figure 6C). Taken together, these data indicate that 12-LOX did not appear to be required for cytokine-induced polarization of macrophages.

**Figure 5:**
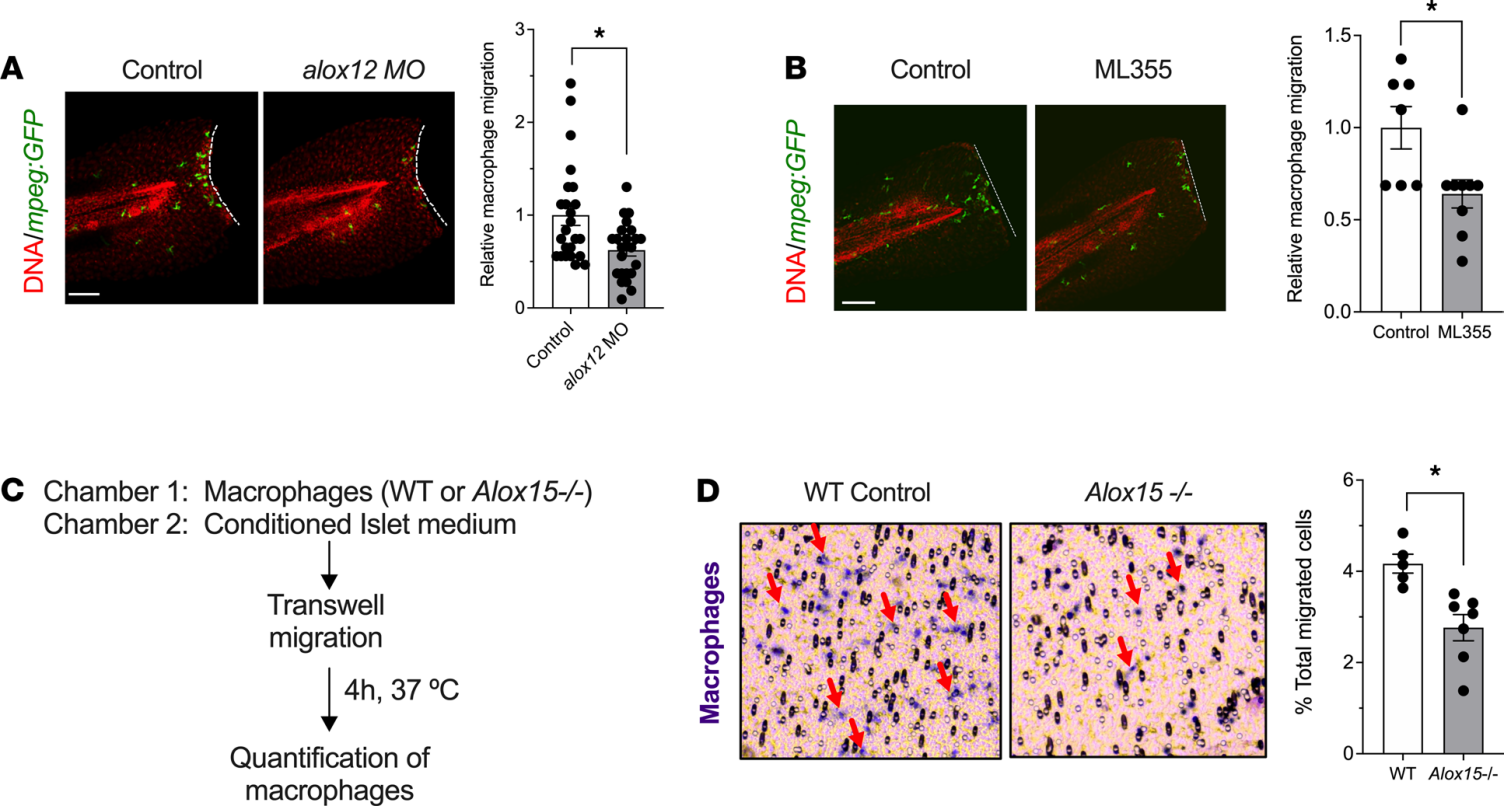
Depletion or inhibition of 12-LOX impairs macrophage migration in zebrafish and mice. (**A**) Control and *alox12* MO-treated *Tg*(*mpeg:GFP*) zebrafish underwent tailfin injury at 3 dpf. Representative images of injured zebrafish tails stained with GFP (macrophages, green) and TO-PRO3 (nuclei, red) are shown on the left and quantitation of relative number of migrating macrophages is shown on the right (*n* = 22–24 fish per condition; *P < 0.05 by unpaired 2-tailed *t* test). (**B**) *Tg(mpeg:eGFP)* zebrafish were treated with vehicle or 10 μM ML355 and then underwent tailfin injury at 3 dpf. Representative images of injured zebrafish tails stained with GFP (macrophages, green) and TO-PRO3 (nuclei, red) are shown on the left and quantitation of relative number of migrating macrophages is shown on the right (*n* = 7–9 fish per condition; *P < 0.05 by unpaired 2-tailed *t* test). (**C**) Description of the chemotaxis assay in vitro using WT and *Alox15^–/–^* mouse peritoneal macrophages. (**D**) Representative images of the porous membrane showing migrating methylene blue–stained macrophages (red arrows) is shown on the left, and quantitation of the number of migrating macrophages is shown on the right (*n* = 5–7 independent experiments; *P < 0.05 by unpaired 2-tailed *t* test). Scale bar: 50 μm. All data are presented as mean ± SEM. 12-LOX, 12-lipoxygenase; *Tg(mpeg:eGFP*), transgenic fish containing enhanced GFP–labeled macrophages; GFP, green fluorescent protein.

Next, we addressed if 12-LOX is required for the ability of cells of the myeloid lineage to migrate. Migration is a critical factor in the ability of myeloid cells to carry out immune surveillance, locate to sites of injury, and present antigen. We first leveraged the tailfin injury model in *Tg*(*mpeg:eGFP*) transgenic zebrafish. *alox12* MO and control fish underwent tailfin injury at 3 dpf, and the number of macrophages migrating to the site of injury was quantitated. In control fish, we observed the expected migration of macrophages to the site of injury within 6 hours, whereas in *alox12* MO fish, there was a significant 38% reduction in the number of macrophages at the site of injury (Figure 5A). As an alternate approach, and to confirm that this finding requires the catalytic activity of 12-LOX, we performed a similar experiment using the well-characterized small molecule 12-LOX inhibitor ML355 (28). For this experiment, we pretreated fish for 2 hours with either vehicle or 10 μM ML355, and then performed tailfin injury. Similar to what we observed with the MO knockdowns, ML355 treatment significantly decreased macrophage migration toward injured sites by 37% as compared with vehicle-treated control embryos (Figure 5B).

To verify that our findings were specific to 12-LOX in macrophages and to confirm their applicability to mammals, we isolated peritoneal macrophages from both *Alox15^–/–^* mice and their littermate controls and performed migration assays in vitro using Transwell chambers. To mimic conditions seen in T1D, we added conditioned culture media from proinflammatory cytokine-treated (IL-1β, IFN-γ, TNF-α) mouse islets to induce migration (Figure 5C). As shown and quantitated in Figure 5D, we observed a significant reduction in the number of *Alox15^–/–^* peritoneal macrophages that transited the Transwell membrane compared with WT control macrophages. These results, consistent with the zebrafish studies, indicate that 12-LOX in macrophages contributed to their ability to migrate under tissue damage/inflammatory conditions.

### Chemokine receptor CXCR3 lay downstream of 12-LOX activity and may have been dependent on the leukotriene B_4_ receptor 2.

**Figure 6:**
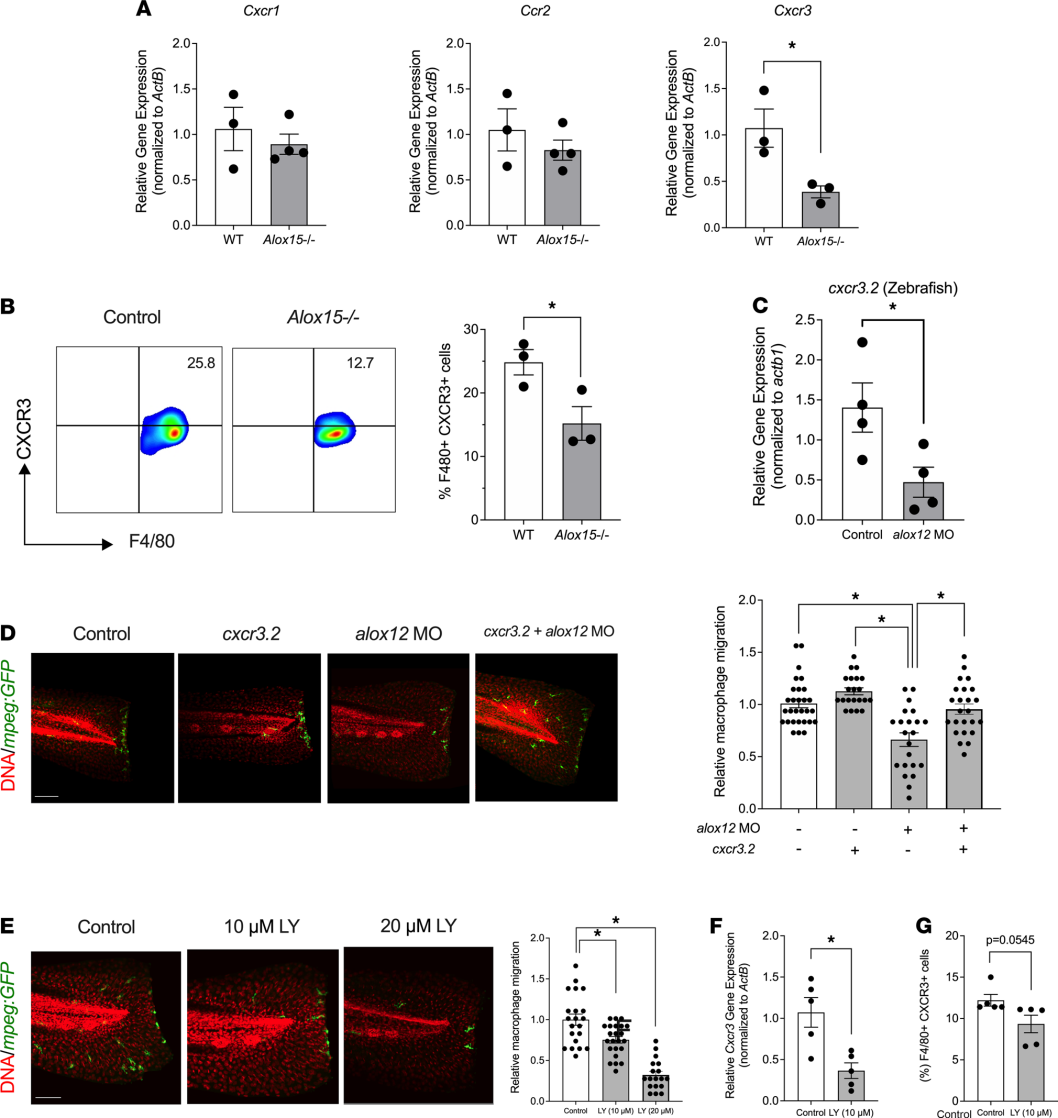
CXCR3 is reduced in the absence of 12-LOX. (**A**) Chemokine receptor mRNA expression in macrophages from WT and *Alox15^–/–^* mice (*n* = 3–4 mice per genotype; *P < 0.05 by unpaired 2-tailed *t* test). (**B**) Representative contour plots showing gating of F4/80^+^ CXCR3^+^ cells are shown on the left and F4/80^+^ CXCR3^+^ cells as a percentage of total peritoneal cells from WT and *Alox15^–/–^* mice are shown on the right (*n* = 3 mice per genotype; *P < 0.05 by unpaired 2-tailed *t* test). (**C**) *cxcr3.2* mRNA expression from whole lysates of control-treated and *alox12* MO-treated zebrafish (*n* = 4 independent experiments from pooled zebrafish larvae; *P < 0.05 by unpaired 2-tailed *t* test). (**D**) Representative images of injured *Tg*(*mpeg:eGFP*) zebrafish tailfins stained with GFP (macrophages, green) and TO-PRO3 (nuclei, red) are shown on the left; and corresponding quantitation of relative number of macrophages at the tail injury site in the control, *alox12* MO–injected fish, fish injected with a vector for macrophage-specific overexpression of *cxcr3.2,* and fish coinjected with *alox12* MO and vector for macrophage-specific overexpression of *cxcr3.2* is shown on the right (*n* = 20–30 fish per condition; *P < 0.05 by 1-way ANOVA with post hoc Tukey’s test). (**E**) Representative images of injured *Tg*(*mpeg:eGFP*) zebrafish tailfins stained with GFP (macrophages, green) and TO-PRO3 (nuclei, red) are shown on the left, and corresponding quantitation of relative number of macrophages at the tail injury site in the vehicle control and BLT2 inhibitor LY255283-treated fish is shown on the right (*n* = 20–25 fish per condition; *P < 0.05 by 1-way ANOVA with post hoc Tukey’s test). Scale bars in **D** and **E** indicate 100 μm. (**F**) *Cxcr3* mRNA expression in mouse peritoneal cells treated with 10 μM LY255283 for 24 hours (cells from *n* = 5 mice per condition; *P < 0.05 by unpaired 2-tailed *t* test). (**G**) F4/80^+^CXCR3^+^ cells as a percentage of total peritoneal cells by flow cytometry after treatment with 10 μM LY255283 for 24 hours (cells from *n* = 5 mice per condition; *P < 0.05 by unpaired 2-tailed *t* test). Data are presented as mean ± SEM. 12-LOX, 12-lipoxygenase; MO, morpholino; GFP, green fluorescence protein; *Tg(mpeg:eGFP*), transgenic fish containing eGFP-labeled macrophages.

Prior studies suggest that 12-LOX and its product 12-HETE alters chemokine receptor expression (34–36). We hypothesized that the macrophage migratory defect we observed in the absence of 12-LOX might be attributable to the loss of 1 or more of these chemokine receptors. We measured the mRNA expression of chemokine receptors *Cxcr1, Ccr2,* and *Cxcr3* implicated in macrophage migration in peritoneal macrophages from *Alox15^–/–^* and control littermate mice. Of the receptor-encoding mRNAs examined, only *Cxcr3* levels were significantly reduced in *Alox15^–/–^* macrophages (Figure 6A). Flow cytometry of peritoneal macrophages confirmed that the cell surface expression of CXCR3 protein was significantly reduced in *Alox15^–/–^* macrophages compared with WT controls (Figure 6B).

To interrogate if the loss of CXCR3 accounts, at least in part, for the defect in macrophage migration observed in the absence of 12-LOX, we returned to zebrafish. As shown in Figure 6C, 12-LOX depletion in *alox12* MO zebrafish resulted in a similar loss of expression of the zebrafish ortholog *cxcr3.2*. Next, we transgenically expressed the *cxcr3.2* coding sequence specifically in zebrafish macrophages (under control of the *mpeg* promoter) to determine if reexpression of this gene was sufficient to rescue the migratory defect in *alox12* MO fish. As shown and quantitated in Figure 6D, *alox12* MO fish injected with a noncoding control vector exhibited the expected reduction in macrophage migration in the tailfin injury assay compared with controls. Importantly, the injection of the *cxcr3.2*-containing vector independently did not alter macrophage migration. However, *alox12* MO fish injected with the *cxcr3.2*-containing vector showed complete rescue of macrophage migration. These results suggest that the reduction of *cxcr3.2* in 12-LOX–depleted zebrafish likely accounted for the defect in macrophage migration.

Finally, we asked if the 12-LOX–dependent migration of macrophages is linked to its eicosanoid product, 12-HETE. The leukotriene B_4_ receptor 2 (BLT2) was previously identified as a low-affinity receptor for 12-HETE (37). Figure 6E shows that *Tg(mpeg:eGFP)* fish treated with an inhibitor of BLT2, LY255283 (38), exhibit a dose-dependent reduction in macrophage migration to the site of tail injury compared with vehicle-treated fish. Furthermore, we also observed that treatment of mouse peritoneal macrophages with LY255283 reduced the expression of the *Cxcr3* gene by qPCR as well as protein levels of CXCR3 by flow cytometry (Figure 6, F and G). These results, which parallel the findings seen in *alox12* MO fish, appear to have linked the dependence of macrophage migration on the 12-HETE receptor BLT2, although further studies are needed to verify this dependency.

## Discussion

In this study, we leveraged the power of a lower model organism and applied these observations to an established mammalian model of T1D to interrogate the participation of myeloid-derived cells in the pathogenesis of insulitis and diabetes progression. Cells of myeloid lineage give rise to a host of circulating cell types, most notably cells of the innate immune system that include monocytes, macrophages, and antigen-presenting DCs. Although such cells have been implicated in the pathogenesis of T1D, and prior studies have attempted myeloid-specific deletions in T1D mouse models (39), we believe our findings are the first to directly demonstrate how genetic manipulation of a signaling pathway specifically in myeloid cells can alter T1D pathophysiology. Key findings from our study suggest (a) that macrophages are active participants in β cell dysfunction and loss in models of both islet injury and autoimmunity; (b) that 12-LOX establishes a prodiabetogenic phenotype of macrophages and DCs that subsequently affects the nature of insulitis and eventual susceptibility to T1D; and (c) that the *Tg*(*ins:NTR*) zebrafish transgenic line provides a highly genetically manipulable platform wherein crosstalk between macrophages and β cells can be modeled and vetted.

Islet-resident myeloid cells have been implicated in the pathogeneses of both T1D and T2D (14, 40). In NOD mice, prior studies have emphasized the role of such resident cells as initiators of the autoimmune response, owing perhaps to their role as antigen-presenting cells. These studies focused on the depletion of macrophages, using either clodronate liposomes (11, 13, 15, 16) or neutralization of a receptor (CSF1 receptor) promoting their development (14). By contrast, our studies did not deplete such cells in total, but rather reduced a subset that appeared to be proinflammatory in nature. Studies of Unanue and colleagues (14) demonstrated that the delivery of a neutralizing antibody against the CSF1 receptor at 3 weeks of age in NOD mice results in the depletion of islet-resident macrophages and is accompanied by a reduction in CD4 T cells and DCs in the insulitic milieu and protects against diabetes. These results align with ours, in which the deletion of *Alox15* in macrophages on the NOD background resulted in significant reduction in insulitis, CD4 T cells, and protection from diabetes in both male and female mice. Moreover, our findings in vivo that myeloid-specific depletion of *Alox15* reduced proinflammatory DCs and enhanced the expression of the mRNA encoding the immune checkpoint protein PD-L1 suggest that the effects we observed may have been related to altered antigen presentation and/or adaptive immune cell activation, respectively. Collectively, our results emphasize that early intervention in the activity of myeloid cells imparted a disease-modifying effect in T1D and, more importantly, that the depletion of the islet-resident populations of these cells was not required to achieve this effect.

An important finding in our studies is the identification that 12-LOX controlled a signaling pathway that affected chemokine receptor expression in macrophages. The gene encoding 12-LOX (*Alox15*) is expressed in islet β cells and macrophages, but not T cells or B cells of the adaptive immune system (41). Although *Alox15* deletion was previously shown to protect against T1D in NOD mice (19), it has remained unclear if the effect could be attributed to its expression in the islets, myeloid cells, or both, particularly since proinflammatory cytokines and their signaling are affected by the loss of *Alox15* in both cell types (30, 41). Our studies in zebrafish provided evidence for a defect in macrophage function upon loss of the zebrafish ortholog using a specific MO because macrophage entry into the islet and subsequent engulfment of β cells appeared defective in *alox12* morphants upon MTZ injury. We acknowledge that MOs can have significant off-target effects; however, here we used a previously validated MO that has been demonstrated to specifically reduce the accumulation of products of 12-LOX, but not products of related 15-LOX or 5-LOX (27). Although it is tempting to speculate that the phenotype in these morphants was attributable to the loss of *alox12* in macrophages, our studies of conditional *Alox15* deletion in myeloid-derived cells in NOD mice proved more conclusive in this regard. Importantly, whereas prior studies of *Cre* recombinase expression on the NOD background leave some doubt as to the effect of the deletion versus the misexpression of *Cre* recombinase on diabetes outcome (42), here we rigorously show that the inclusion of littermate controls containing either the homozygous *Loxp* alleles or *Cre* recombinase developed T1D at the expected frequencies in both sexes. Therefore, we believe that the remarkable protective phenotype of NOD mice harboring myeloid cell–specific loss of the *Alox15* alleles was directly attributable to the loss of 12-LOX activity in these cells.

The mechanism by which 12-LOX promotes T1D progression has been variably attributed to cytokine signaling, oxidative stress, and cellular apoptosis induced by its major arachidonic acid–derived eicosanoid 12-HETE (17). The majority of these prior studies were performed in islets and β cell–derived cell lines. Here, we demonstrate mechanisms by which 12-LOX impacted myeloid cell function in T1D. We show that 12-LOX was not required for the apparent polarization of macrophages to proinflammatory or antiinflammatory states, suggesting that its effects may be more specific to functional duties commonly ascribed to myeloid cells. The chemokine receptor CXCR3 and its ligands CXCL9, CXCL10, and CXCL11 serve as part of the chemoattractant response during inflammation and immunity. Here and elsewhere (43), CXCR3 has been shown to be expressed on myeloid-derived cells and may be required for local migration for immune surveillance, antigen presentation, and tissue damage clearance.

Studies on the role of CXCR3 in the context of NOD mice have shown conflicting results, with some suggesting that its deficiency protects against diabetes (44) and others suggesting its deficiency accelerates diabetes (45). A complexity in these prior studies is the global nature of deletion and the differences in diabetes induction in these animal models. Although our studies do not entirely resolve this issue, they do indicate a critical role of this receptor for a fundamental function of myeloid cells. We show that 12-LOX was required for cellular migration, likely through the expression of CXCR3 in mice and its ortholog CXCR3.2 in zebrafish, where the reexpression of *cxcr3.2* specifically in macrophages restored the migratory capacity. That the migratory defect can be rescued by transgenic reexpression emphasizes that the effect of 12-LOX was exerted at the transcriptional level rather than the posttranscriptional level. This finding is especially relevant because 12-LOX is an enzyme with no known transcriptional function per se. It remains possible that this transcriptional effect was the result of the downstream activity of BLT2, a receptor for 12-HETE. Further studies of mutant zebrafish and mice will be required to address this issue.

A final major implication and limitation of our study is the relevance of zebrafish. We present a model system in which macrophages play a central role in destruction of β cells in a transgenic *Tg*(*ins:NTR*) line of zebrafish. In the absence of adaptive immune cell involvement in our zebrafish model, it would be inaccurate to claim that this system models T1D or that it can be used to study relevant agents that might be used to treat T1D. Nevertheless, this model system exhibits some features that allow its use as a platform for the study the dynamics between macrophages and β cells. We showed that the depletion of macrophages in zebrafish using clodronate preserved preexisting β cells after MTZ treatment of zebrafish, yet prevented the formation of neogenic β cells—the combined effect of which may have resulted in only the modest effect on glycemia that we observed. On the one hand, these findings reflect similar studies in NOD mice in which clodronate treatment preserved β cells and glycemia (11, 16), and, on the other, they support studies in mice that macrophages promote β cell proliferation and tissue regeneration (46, 47). These seemingly dichotomous findings likely reflect the differential involvement of proinflammatory (“M1”) and antiinflammatory (“M2”) macrophages, which are not differentially selected by clodronate treatment. Our studies with *alox12* MO and the 12-LOX inhibitor ML355 appears to target the “prodiabetogenic” (presumably M1-like) macrophages, findings that are supported by our tissue-specific KO studies in NOD mice. In ongoing studies, our laboratory is interrogating the potential existence of different macrophage phenotypes in zebrafish, the implications of which would broaden further the applicability of zebrafish models for interrogating innate immune-mediated tissue injury and repair.

**Figure 7:**
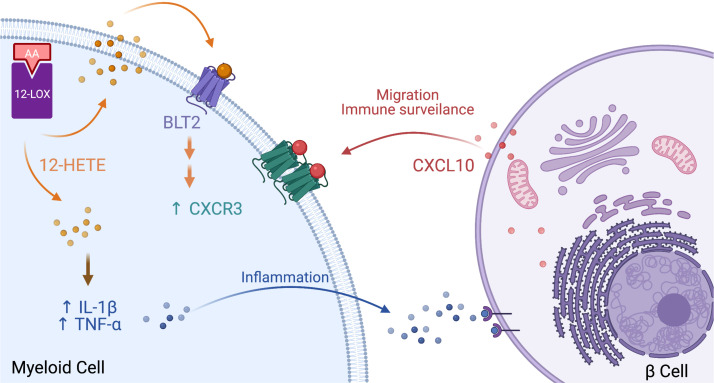
Model for 12-LOX regulation of macrophage inflammation, migration, and surveillance. The model shown depicts a myeloid cell (left) and an islet β cell (right), and the proposed role of 12-LOX in myeloid cells during the pathogenesis of type 1 diabetes. The activity of 12-LOX catalyzes the production of 12-HETE from membrane phospholipid–derived AA. Elevations in 12-HETE levels result in increased production of proinflammatory cytokines (e.g., IL-1β, TNF-α) and, through activation of BLT2, enhanced expression of CXCR3; these effects of 12-HETE lead, respectively, to islet β cell inflammation and enhanced migration and immune surveillance. Image created with BioRender. 12-LOX, 12-lipoxygenase; 12-HETE, 12-hydroxyeicosatetraenoic acid; AA, arachidonic acid.

In conclusion, our findings support a role for macrophages and DCs in the initiation of T1D and, more importantly, implicate a central role for 12-LOX in promoting the initial innate immune response during diabetes pathogenesis. Figure 7 shows a schematic representation of the findings of our study in the context of the DC–β cell interactions that govern the pathophysiology of T1D. We propose that 12-LOX in proinflammatory macrophages and DCs promotes the expression of *Cxcr3* (possibly via BLT2) to permit cellular migration, antigen acquisition and presentation, and production of proinflammatory cytokines (IL-1β, TNF-α). The deletion or inhibition of 12-LOX in the early phases of T1D impairs these processes, thereby reducing antigen acquisition and presentation, insulitis, and β cell loss. This model does not explicitly exclude an independent role for 12-LOX in β cells, where it promotes proinflammatory signaling and leads to cellular stress and apoptosis. Whether the deletion of *Alox15* in β cells might independently disrupt this interaction between cells remains an ongoing study in our laboratory. Some limitations of our study are worth noting. First, our study deleted 12-LOX from the inception of *Lyz2* expression in all myeloid cells during mouse development; hence, it remains unclear (a) if its role beyond macrophages and DCs (such as neutrophils) might have also contributed to the phenotype observed, and (b) if the loss of *Alox15* during myeloid cell development might have inherently prevented the acquisition of a prodiabetogenic phenotype before even the initiation of the disease process. The latter point could have implications for the timing of targeted therapeutics. Second, as noted above, our studies in zebrafish represent only a platform to interrogate mechanisms but cannot be used to infer disease pathogenesis. Ongoing studies in the lab are focused on the timing of *Alox15* deletion relative to disease onset and the translation of these studies to human disease and humans.

## Methods

### Zebrafish and mouse strains and maintenance.

Zebrafish (*D*. *rerio*) were maintained at 28.5°C in a recirculating aquaculture system enclosed in a cabinet and subjected to a 14-hour light/10-hour dark cycle. The following transgenic lines (originally obtained through the Zebrafish International Resource Center, Eugene, OR) were used in the experiments: *Tg*(*ins:NTR*)*^s950^* (48), *Tg*(*mpeg1:eGFP*)*^gl22^* (22), and *Tg*(*ins:Kaede*)*^s949^* (49). Heterozygous outcrossed embryos bearing relevant transgenic alleles were collected at spawning and maintained in a 28.5°C incubator in Petri dishes with buffered egg water (0.1% instant ocean salt, 0.0075% calcium sulfate supplemented with 0.003%1-Phenyl-2-thiourea; Acros) to prevent pigmentation in all embryos. At 3 dpf (larval stage), the transgenic zebrafish were genotyped by epifluorescence using a Leica M205FA dissecting microscope. MOs were purchased from Gene Tools LLC. The *alox12* MO targeting the translational start were described previously (27), and the control (nontargeting) MO sequence was 5′-CCTACCTCAGTTACAATTTATA-3′. A *cxcr3.2*-containing expression vector was generated by inserting the coding sequence of *D*. *rerio* *cxcr3.2* gene under control of the *D*. *rerio* macrophage-specific *mpeg* promoter (*mpeg:cxcr3.2*).

All mouse experiments were performed under specific pathogen–free conditions. *Alox15^+/–^* mice on the *C57BL/6J* background were purchased from Jackson Laboratories and maintained and bred in-house. For the experimental controls, we utilized WT littermates from breedings. *C57BL/6J.Alox15^loxP/loxP^* (29, 30) and *C57BL/6J.129P2-Lyz2^tm1(cre)lfo^/J* (*Lyz2-Cre*) were backcrossed onto the *NOD.ShiLt/J* background using speed congenics services provided through Jackson Laboratories. After successful backcrossing, *NOD.Alox15^loxP/loxP^* mice were crossed with *NOD.Lyz2-Cre* mice to generate breeding colonies. All controls for NOD mouse experiments were littermates (either *NOD.Alox15^loxP/loxP^* or *NOD.Lyz2-Cre*). Diabetes incidence was monitored as described previously (50).

### Zebrafish manipulations.

For the β cell injury assay, zebrafish were washed with egg water and then treated with 7.5 mM MTZ (MilliporeSigma) prepared in egg water or egg water alone as described previously (23). After MTZ treatment, fish were washed with egg water and allowed to recover for the times indicated. For the tailfin injury assay, fish were transiently paralyzed with 0.01% tricaine (MilliporeSigma) in egg water to restrict their movements, and the distal tip of the tail fin was amputated with a scalpel. At the end of each experiment, fish were fixed, deyolked, and immunostained as previously described (51). The following concentrations of primary antibodies were used: 1:200 guinea pig anti-insulin (Invitrogen) and 1:200 chicken anti-GFP (Aves Labs). Primary antibodies were detected with 1:500 dilutions of complementary Alexa-conjugated secondary antibodies (Jackson ImmunoResearch). DNA was stained with 1:500 TO-PRO3 (Thermo Fisher). After staining, fish were mounted on charged glass slides in VECTASHIELD (Vector Labs) and confocal imaging was performed with an LSM800 microscope (Zeiss) or an A1 microscope (Nikon). For macrophage depletion studies, fish were first sedated with 0.01% tricaine, mounted in 2.5% methylcellulose (Electron Microscopy Sciences), and then injected transpericardially with 7–10 nL clodronate liposomes or control liposomes (Encapsula Nano Sciences) 24 hours prior to experimentation. For inhibitor studies, fish were pretreated with 12-LOX inhibitor (ML355), BLT2 inhibitor (LY255283), or vehicle (0.1% DMSO) for 2 hours.

For measurement of glucose levels, whole fish were homogenized in 500 μL glucose assay buffer provided in the Glucose Colorimetric Assay Kit (Biovision). Samples were then centrifuged at 2000*g* for 5 minutes at room temperature, and the supernatant was used in duplicate for the glucose assay following the manufacturer’s protocol. The colorimetric assay was measured using a SpectraMax iD5 multimode microplate reader (Molecular Devices) at 405 nm.

### IHC and β cell mass.

Pancreata from at least 5 different mice per group were fixed in 4% paraformaldehyde, paraffin embedded, and sectioned onto glass slides. Pancreata were immunostained using rabbit anti-insulin (1:1000; ProteinTech). β Cell mass was calculated as previously detailed (52). Insulitis was scored as previously described (50) using the following scoring criteria: 1, no insulitis, 2, infiltrate less than 50% circumference, 3, infiltrate greater than 50% circumference, 4, infiltration within the islet.

### Primary cell isolations, incubations, and analyses.

Islets from mice were isolated as previously described (53). Mouse peritoneal macrophages were isolated as described (54) immediately after euthanasia by injecting ice-cold RPMI into the peritoneal cavity using a 25-gauge needle. The injected RPMI was then removed. The isolated cells were lysed with RBC lysis buffer (eBioscience) to remove red blood cells. Naive T cells were isolated and purified from spleen and lymph nodes of *C57BL/6J* mice using EasySep Mouse CD4^+^ T Cell Isolation Kit (STEMCELL Technologies). Pancreata were dissected from mice and incubated with 1 mg/mL Collagenase P solution without serum at 37°C for 15–30 minutes, then dissociated by passage through 18-gauge needles prior to flow cytometry analysis.

For polarization studies in vitro, isolated peritoneal macrophages stimulated with 10 ng/mL LPS and 25 ng/mL IFN-γ (for M1 polarization), 10 ng/mL IL-4 (for M2 polarization), or media control for 16 hours. Peritoneal macrophages from NOD mice were treated with 100 ng/mL PMA (MilliporeSigma), 500 ng/mL Ionomycin (MilliporeSigma), and Golgi Stop Plug (1:1000; BD Pharmingen) prior to immunostaining. To stain for surface antigens, cells were incubated with antibodies against F4/80 (BM-8, BioLegend), CD11c (HL3, BD Pharmingen), CD4 (RM4-5, BioLegend), CD80 (16-10A1, BioLegend), CD86 (GL-1, BioLegend), CD45 (30-F11, BD Biosciences), and/or MHC-II (M5/114.15.2, BioLegend) or the appropriate isotype controls for 30 minutes. For cytokine staining, the cells isolated were stimulated with 100 ng/mL PMA (MilliporeSigma), 500 ng/mL Ionomycin (MilliporeSigma), and Golgi Stop Plug (1:1000; BD Pharmingen). Then cells were permeabilized using Cytofix/Cytoperm (BD Pharmingen) and incubated with antibodies for TNF-α (BioLegend), IL-1β (Thermo Fisher), IL-17 (TC11-18H10, BD Pharmingen), IFN-γ (XMG12, BD Pharmingen), and/or Foxp3 (MF23, BD Pharmingen). All antibodies were used at 1:100 dilution. Cells were filtered and acquired on the Attune NxT Flow Cytometer or a FACSCanto II cytometer (BD Biosciences). Data were analyzed using FlowJo software (Tree Star). Supernatant from stimulated peritoneal macrophages was collected and IL-6 and IL-12 levels were measured by ELISA according to the manufacturer’s instructions (eBioscience) and read on a SpectraMax iD5 multimode microplate reader (Molecular Devices).

### qPCR analysis.

RNA was isolated from zebrafish or from mouse tissue using the RNeasy Plus Micro Kit (QIAGEN) and was used to prepare cDNA using a commercial kit (Applied Biosystems). qPCR was run using the SsoFast EvaGreen Supermix Kit (Bio-Rad) on a Quantstudio 3 thermocycler (Applied Biosystems). The average Ct value of 3 replicates was calculated and normalized to β-actin.

### Immunoblot analysis.

Mouse peritoneal cells were treated with 10 μM arachidonic acid for 4 hours at 37°C, then lysed in lysis buffer (Thermo Fisher) and subjected to electrophoresis on a 4%–20% gradient SDS–polyacrylamide gel. Antibodies included rabbit polyclonal antibody directed against 12-LOX (1:1000; Abcam) and mouse monoclonal antibody directed against eIF5A (1:1000; BD Biosciences). Immunoblots were visualized using fluorescently labeled secondary antibodies (LI-COR Biosciences) and were quantified using LI-COR software.

### Transwell chemotaxis assay.

A 96-well chemotaxis system (ChemoTx, 8 μm filter pore size; Neuro Probe) was loaded with conditioned media from islets isolated from *C57BL/6J* mice. Isolated islets were treated with a proinflammatory cytokine cocktail (50 ng/mL TNF-α, 25 ng/mL IL-1β, 100 ng/mL IFN-γ) for 24 hours after isolation to generate conditioned media. The conditioned media were collected and loaded in the bottom chamber of the chemotaxis system. WT or *Alox15^–/–^* macrophages (0.5 × 10^5^ cells) were added to the upper chamber and migration to the lower chamber was measured after incubation for 4 hours at 37°C. After incubation, nonmigrated cells were washed, and the filter side containing the migrated cells was stained with Kwik-Diff solution kit (Shandon, Thermo Fisher). The filter was mounted on the slides, and the number of migrated macrophages was quantified by manual counting under an LSM800 microscope (Zeiss).

### Statistics.

All data are presented as mean ± SEM. Data analyses were performed using the GraphPad Prism 9 software package. Significant differences between the mean values were determined using a 2-tailed Student’s *t* test, where 2 means were compared, and 1-way ANOVA followed by post hoc Tukey’s test when more than 2 means were compared. The differences were considered statistically significant at a *P* value of less than 0.05.

### Study approval.

Experiments involving mice were performed under protocols approved by the IACUCs of the University of Chicago, Indiana University, and Eastern Virginia Medical School. Experiments involving zebrafish were performed under protocols approved by the IACUCs of the University of Chicago and Indiana University.

## Author contributions

AK, JLN, MAM, SAT, RGM, and RMA conceived the study. AK, ARP, IC, MAW, SI, MHP, KSO, LG, MAM, SAT, and RMA provided methodology. AK, ARP, IC, MAW, JLN, MAM, SAT, RGM, and RMA analyzed the data. AK, SAT, RGM, and RMA wrote the original draft. All authors reviewed and edited the manuscript. RGM and JLN acquired the funding. All authors have read and concurred with the final version of the manuscript.

## Supplementary Material

Supplemental data

## Version 1 06/15/2021

In-Press Preview

## Version 2 07/22/2021

Electronic publication
