# Supplementary material for: 12-Lipoxygenase governs the innate immune pathogenesis of islet inflammation and autoimmune diabetes
Source: JCI Insight. 2021 Jul 22;6(14):e147812. doi: 10.1172/jci.insight.147812 (PMC8410073; doi:10.1172/jci.insight.147812)
Supplement: Supplemental data [file jciinsight-6-147812-s070.pdf]

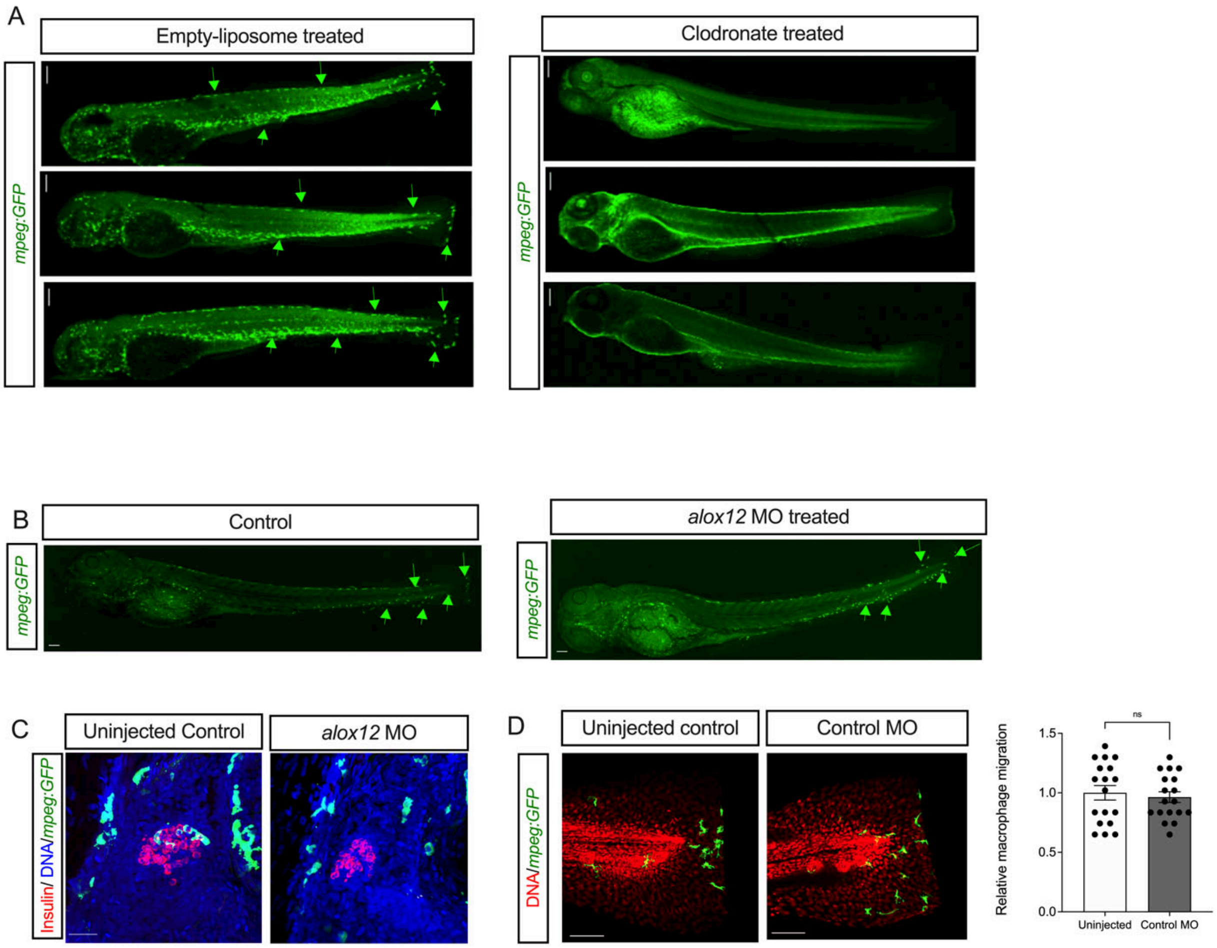

Supplementary Figure S1: Effect of clodronate liposomes and *alox12* MO on whole-embryo macrophages. (***A***) Three representative images of *Tg(mpeg:GFP)* zebrafish injected with empty control liposomes or with clodronate liposomes and stained for GFP (macrophages, green). *Arrows* indicate GFP+ macrophages; (***B***) Representative images of *Tg(mpeg:GFP)* zebrafish in control (uninjected) condition or injected with *alox12* MO. *Arrows* indicate GFP+ macrophages; (***C***) Representative images of islets from *Tg(mpeg:GFP);Tg(ins:NTR)* zebrafish treated with MTZ, then stained for insulin (*β cells, red*), GFP (*macrophages, green*), and DAPI (*nuclei, blue*). (***D***) Representative images of uninjected controls and control MO-injected *Tg(mpeg:GFP)* zebrafish tails after 6 hours of tailfin injury on *left* and quantitation on the *right.* **P*<0.05 by unpaired two-tailed t-test. Scale bars indicate 100 μm.

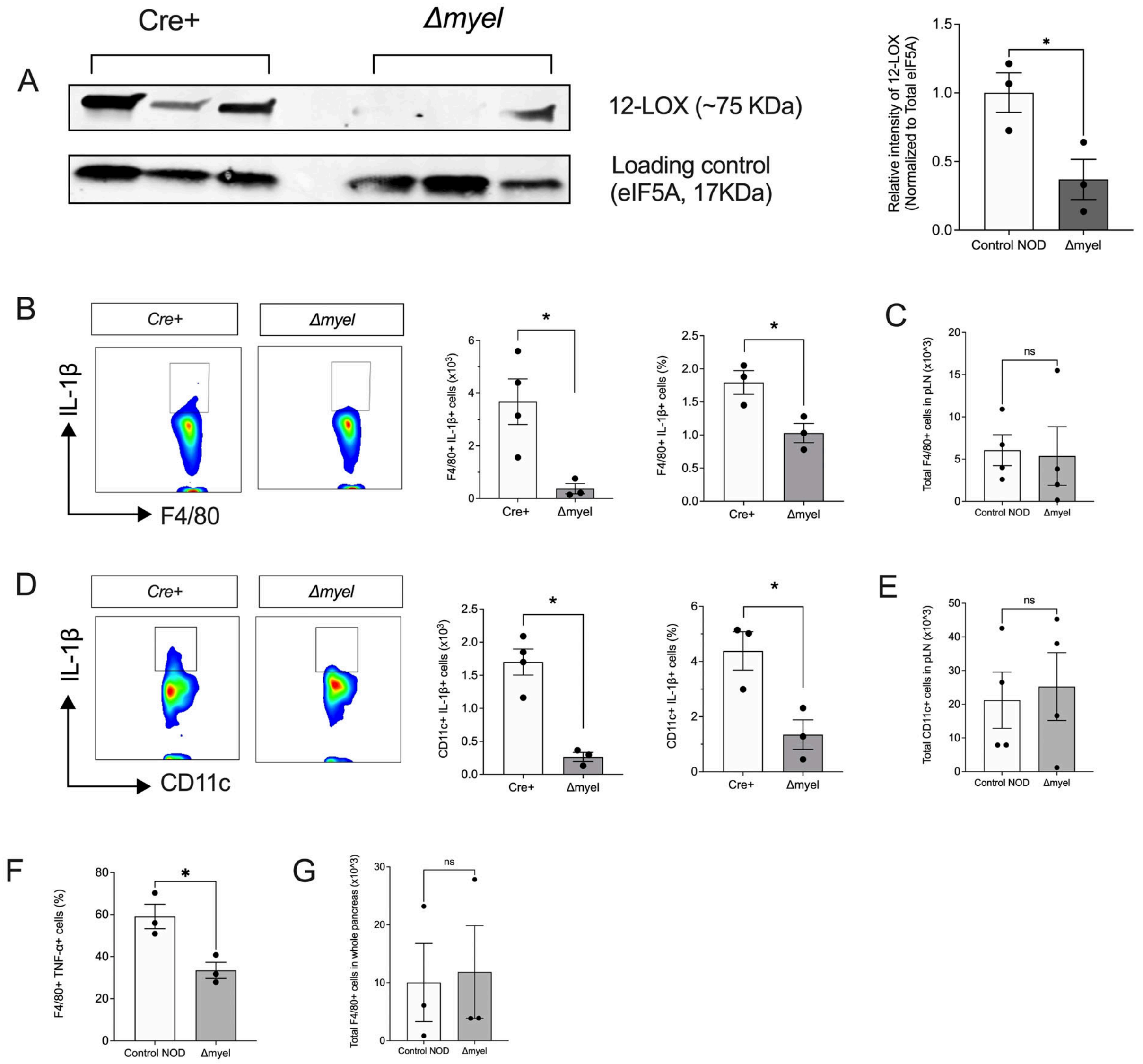

Supplementary Figure S2: Reduced proinflammatory myeloid cell populations in pancreatic lymph nodes of *NOD:Alox15*$^{\Delta myel}$ mice. (***A***) Peritoneal cells were isolated from *NOD:Lyz2-Cre* (*Cre+*) control mice and *NOD:Alox15*$^{\Delta myel}$ (Δ*myel*) mice at 10 weeks of age and subjected to immunoblot analysis (*left*) and quantitation of the relative band intensities of 12-LOX normalized to the band intensities of eIF5A (*right*). **P*<0.05 by unpaired two-tailed t-test; (***B***) Pancreatic lymph nodes were isolated from *NOD:Lyz2-Cre* (*Cre+*) control mice and *NOD:Alox15*$^{\Delta myel}$ (Δ*myel*) mice at 8 weeks of age and subjected to flow cytometry analysis. Representative contour plot showing gating of F4/80+ IL-1β+ cells (*left*), total number of F4/80+ IL-1β+ cells (*middle*), and F4/80+ IL-1β+ cells as a percentage of total cells (*right*). **P*<0.05 by unpaired two-tailed t-test; **(*C*)** Quantitation of total number of macrophages (F4/80+ cells) in the pancreatic lymph nodes of *Cre+* and Δ*myel* mice. **P*<0.05 by unpaired two-tailed t-test; (***D***) Representative contour plot showing gating of CD11c+ IL-1β+ cells (*left*), total number of CD11c+ IL-1β+ cells (*middle*) and CD11c+ IL-1β+ cells as a percentage of total cells (*right*). **P*<0.05 by unpaired two-tailed t-test; (***E***) Quantitation of total number of dendritic cells (CD11c+ cells) in the pancreatic lymph nodes of *Cre+* and Δ*myel* mice. **P*<0.05 by unpaired two-tailed t-test; (***F***) Quantitation of total number of macrophages (F4/80+ cells). **P*<0.05 by unpaired two-tailed t-test; (**G**) proinflammatory macrophages (F4/80+ TNF-α+ cells) in the whole pancreas of *Cre+* and Δ*myel* mice. **P*<0.05 by unpaired two-tailed t-test. All data are presented as mean ± SEM.

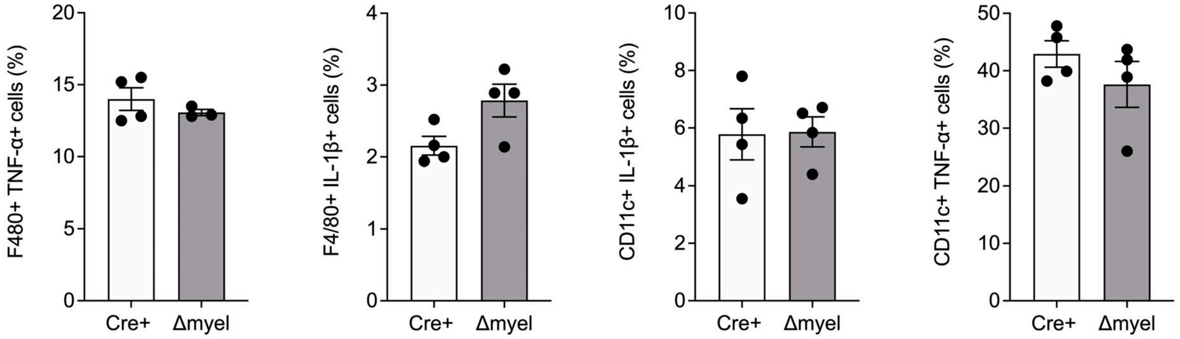

Supplemental Figure S3: Unaltered myeloid cell populations in spleen of control *NOD-Lyz2-Cre* and *NOD:Alox15*$^{\Delta myel}$ mice. Spleens were isolated from control *NOD-Lyz2-Cre* (*Cre*+) and *NOD:Alox15*$^{\Delta myel}$ (Δ*myel*) mice at 8 weeks of age and subjected to flow cytometry analysis for proinflammatory myeloid cell populations. Shown are the F4/80+ IL-1β+ cells, F4/80+ TNFα+ cells, CD11c+ IL-1β+ cells, and CD11c+ TNFα+ cells as a percentage of total cells. Data are presented as mean ± SEM.

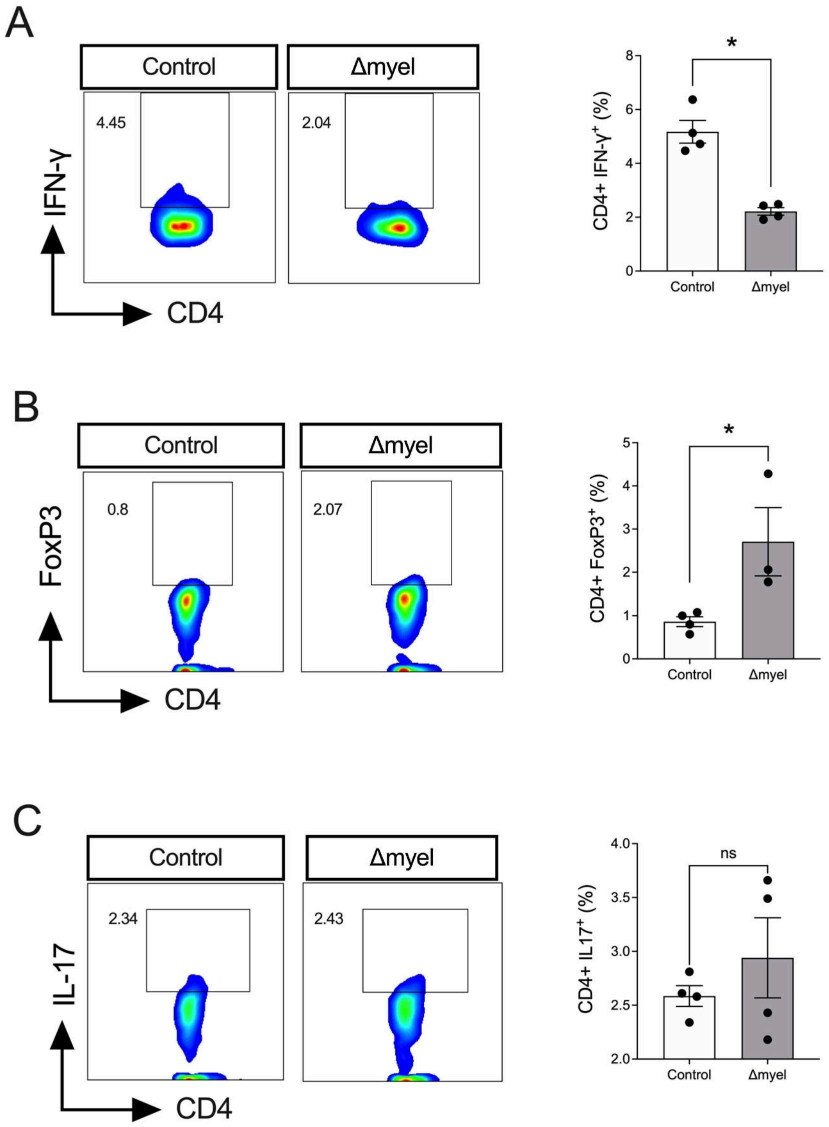

Supplemental Figure S4: Alterations in the T cell population of control *NOD-Lyz2-Cre* and *NOD:Alox15*$^{\Delta myel}$ mice. Pancreatic lymph nodes were isolated from control *NOD-Lyz2-Cre* (*Cre*+) and *NOD:Alox15*$^{\Delta myel}$ (Δ*myel*) mice at 8 weeks of age and subjected to flow cytometry analysis for T cell populations. Shown are the contour plots of (***A***) CD4+IFN-γ+ cells, (***B***) CD4+Foxp3+ cells, and (***C***) CD4+IL-17+ cells on the *left* and the quantitation as a percentage of total cells on the *right.* **P*<0.05 by unpaired two-tailed t-test. Data are presented as mean ±SEM.

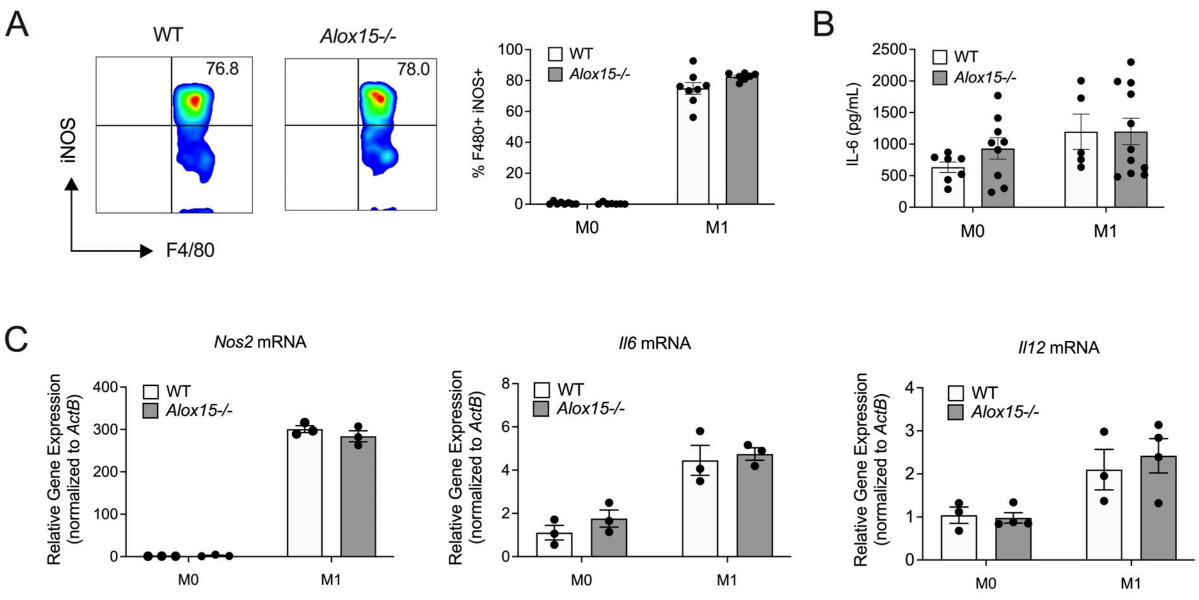

Supplemental Figure S5: Effect of 12-LOX depletion on "M1" polarization of macrophages. Peritoneal cells were isolated from wildtype and *Alox15-/-* mice and unpolarized (M0) or polarized in vitro to a "M1-like" state upon incubation with lipopolysaccharide (LPS) and IFN-γ, then subjected to flow cytometry analysis and quantitative RT-PCR. (***A***) Representative contour plot showing gating of F4/80+ iNOS+ cells is shown on the *left* and quantitation of F4/80+ iNOS+ cells as a percentage of total cells is shown on the *right*; (***B***) IL6 levels in media of unpolarized (M0) and M1-polarized cells; (***C***) quantitative RT-PCR data for the indicated genes (normalized to *Actb*). All data are presented as mean ± SEM. Statistics were evaluated by unpaired two-tailed t-tests for comparison between WT and *Alox15-/-.*

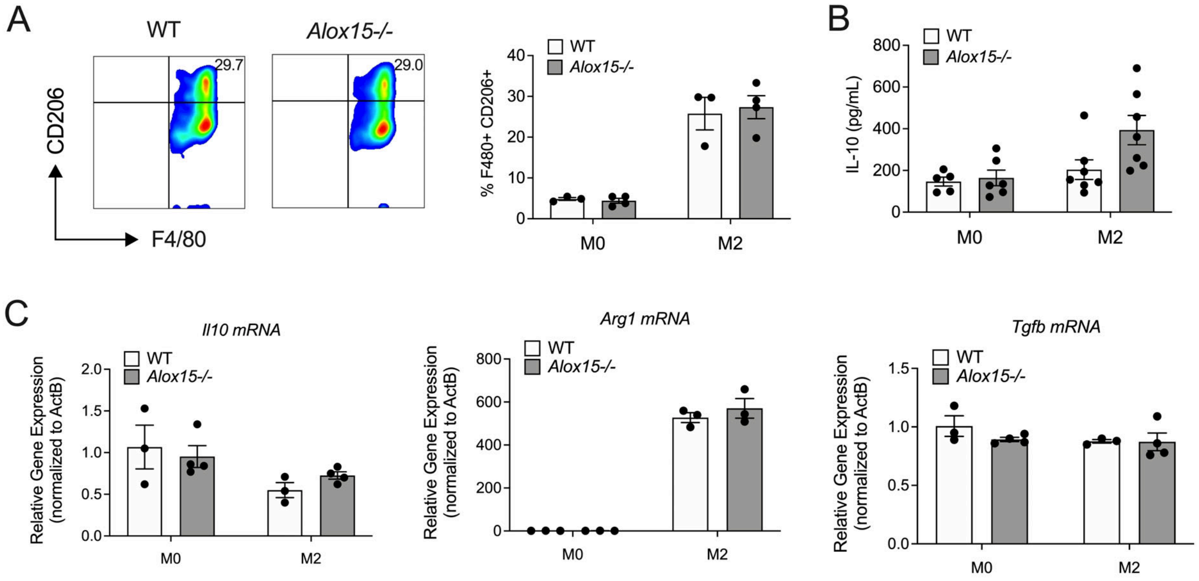

Supplemental Figure S6: Effect of 12-LOX depletion on "M2" polarization of macrophages. Peritoneal cells were isolated from wildtype and *Alox15-/-* mice and unpolarized (M0) or polarized in vitro to a "M2-like" state upon incubation with IL-4, then subjected to flow cytometry analysis and quantitative RT-PCR. (***A***) Representative contour plot showing gating of F4/80+ CD206+ cells is shown on the *left* and quantitation of F4/80+ CD206+ cells as a percentage of total cells is shown on the *right*; (***B***) IL-10 levels in media of unpolarized (M0) and M2-polarized cells; (***C***) quantitative RT-PCR data for the indicated genes (normalized to *Actb*). All data are presented as mean ± SEM. Statistics were evaluated by unpaired two-tailed t-tests for comparison between WT and *Alox15-/-.*
